# Pericarditis Secondary to an Acupuncture Needle Extracted Via a Transjugular Approach

**DOI:** 10.1016/j.jaccas.2021.09.011

**Published:** 2021-12-01

**Authors:** Wassim Bedrouni, Michael Chetrit, Abdullah Al Isma'ili, Christos Galatas, Youri Kaitoukov, Bojan Kovacina, Vartan Mardigyan

**Affiliations:** a Department of Medicine, McGill University Health Center, Montreal, Quebec, Canada; b Department of Medicine, Cité de la Santé Hospital, Laval, Quebec, Canada; c Department of Radiology, Jewish General Hospital, Montreal, Quebec, Canada; d Department of Medicine, Jewish General Hospital, Montreal, Quebec, Canada

**Keywords:** acupuncture, percutaneous intervention, pericardial disease, pericarditis, BP, blood pressure, CMR, cardiac magnetic resonance, CRP, C-reactive protein, IVC, inferior vena cava

## Abstract

Acupuncture is generally considered safe; however, cardiac complications can occur. We describe a case of refractory pericarditis requiring transvenous extraction of an acupuncture needle from within the right ventricular cavity. (**Level of Difficulty: Intermediate.**)

## Central Illustration

**Figure:**
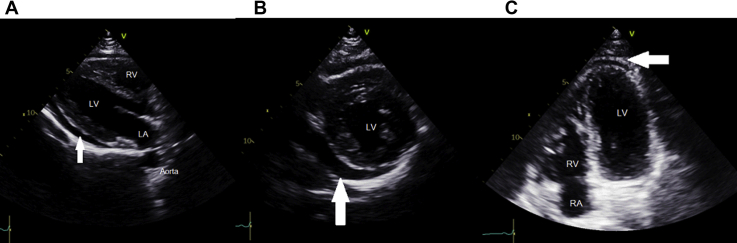

## History of Presentation

**Figure 1:**
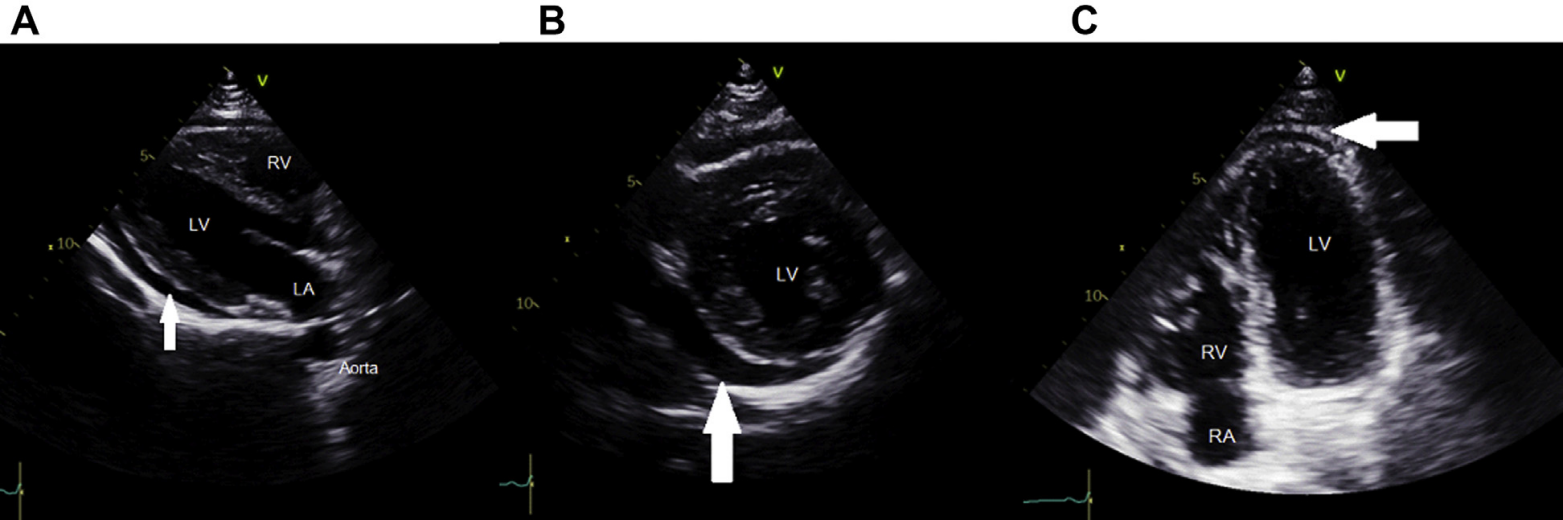
Initial Echocardiography **(A)** Parasternal long-axis, **(B)** parasternal short-axis, and **(C)** off-axis apical views on transthoracic echocardiography showing a small pericardial effusion **(arrows).** LA = left atrium; LV = left ventricle; RA = right atrium; RV = right ventricle.

A 24-year-old man presented to the emergency department with pleuritic chest pain that worsened when he was recumbent. The physical examination was unremarkable, with an initial blood pressure (BP) of 125/86 mm Hg and a pulse of 90 beats/min. The electrocardiogram showed an incomplete right bundle branch block. A transthoracic echocardiogram demonstrated a small pericardial effusion (Figures 1A to 1C, Videos 1A, 1B, and 1C). A clinical diagnosis of acute pericarditis was made, given 2 of 4 diagnostic criteria present, and the patient was sent home on high-dose ibuprofen and colchicine (1).

**Learning Objectives**

• To appreciate that the management of complex pericardial disease often requires a multimodality imaging approach in addition to a thorough history and physical examination.
• To recognize that, although rare, cardiovascular complications of acupuncture can occur.

**Figure 2:**
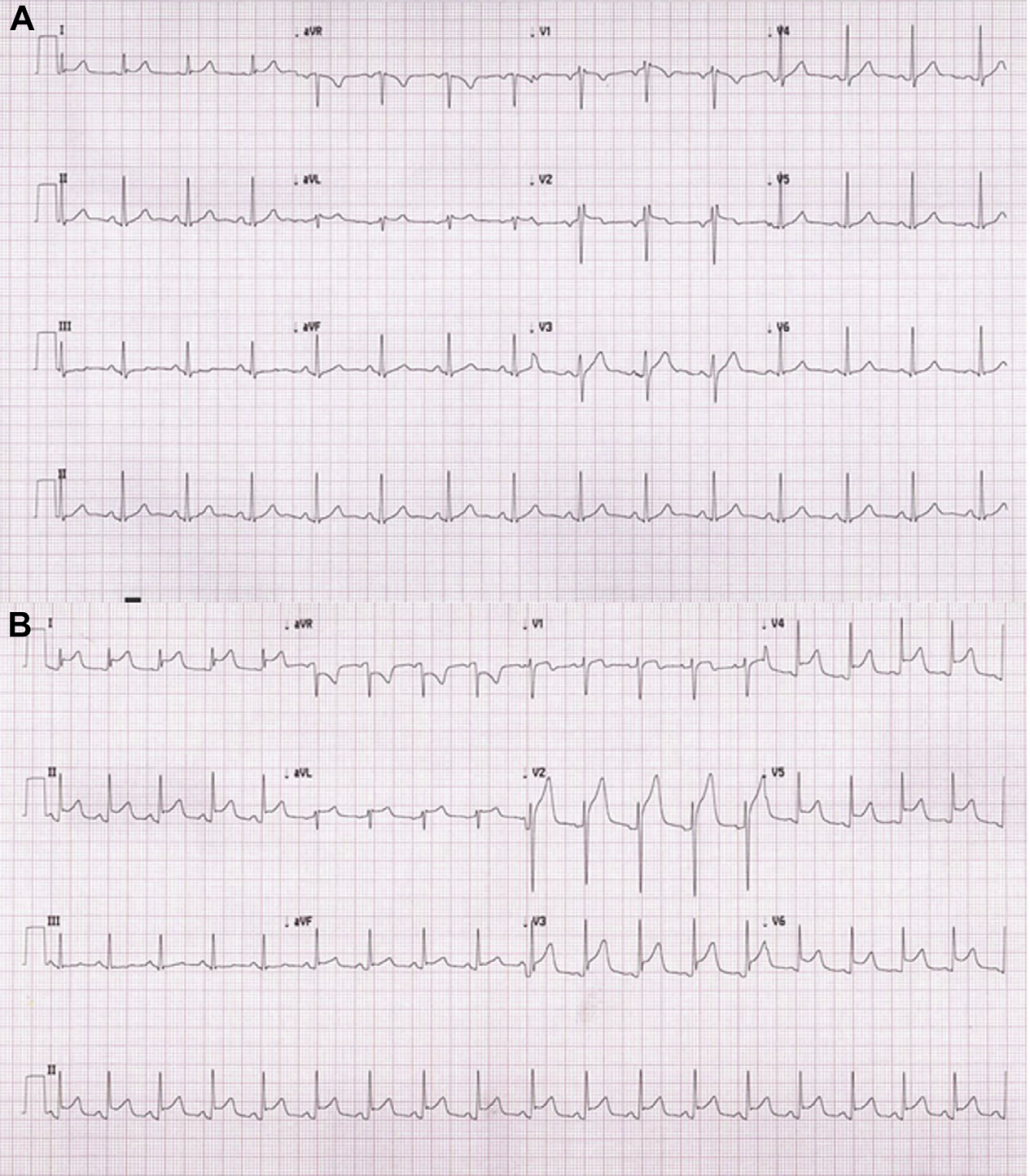
Electrocardiograms on Initial and Repeat Presentation **(A)** Initial 12-lead electrocardiogram showing sinus rhythm. **(B)** Repeat 12-lead electrocardiogram showing sinus tachycardia, diffuse concave ST-segment elevations, and PR depression.

The patient reconsulted 12 hours after discharge with worsened chest pain, vomiting, and dyspnea. The repeat physical examination demonstrated a BP of 101/75 mm Hg, a pulse of 122 beats/min, and jugular venous distention. In contrast to the initial electrocardiogram (Figure 2A), a repeat electrocardiogram showed new, diffuse ST-segment elevation with PR depressions (Figure 2B).

## Past Medical History

This patient reported no past medical history.

## Differential Diagnosis

Pericarditis that progresses rapidly despite anti-inflammatory medications suggests an exaggerated inflammatory response or a nonidiopathic origin.

## Investigations

**Figure 3:**
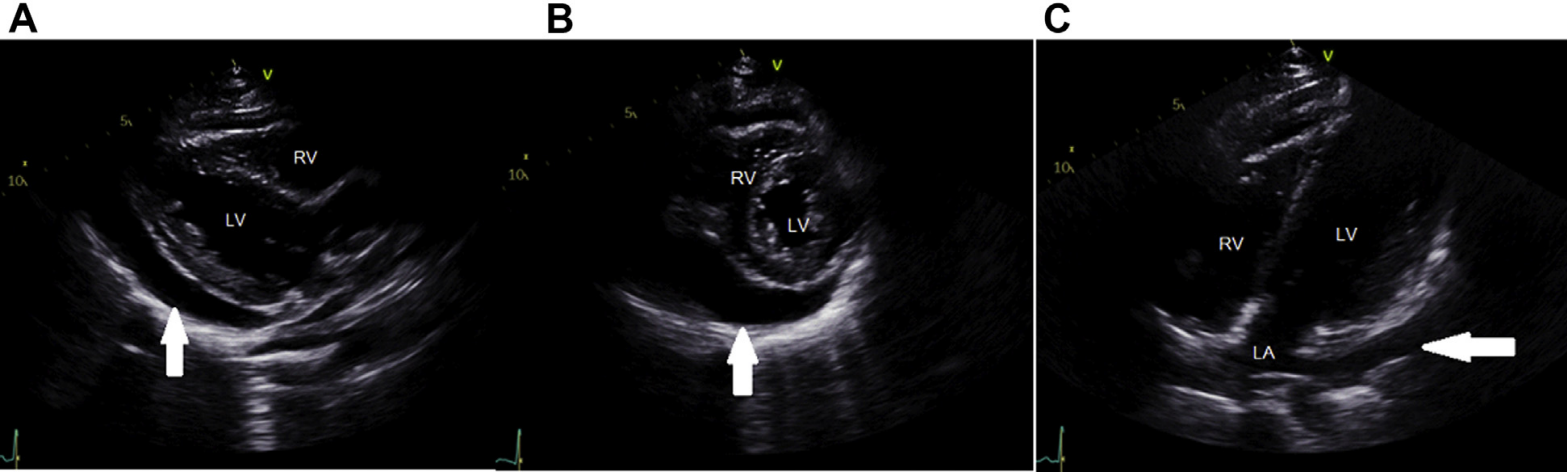
Repeated Echocardiography Repeat **(A)** parasternal long-axis, **(B)** parasternal short-axis, and **(C)** off-axis apical views. The pericardial effusion has increased in size **(arrows).** Abbreviations as in Figure 1.

**Figure 4:**
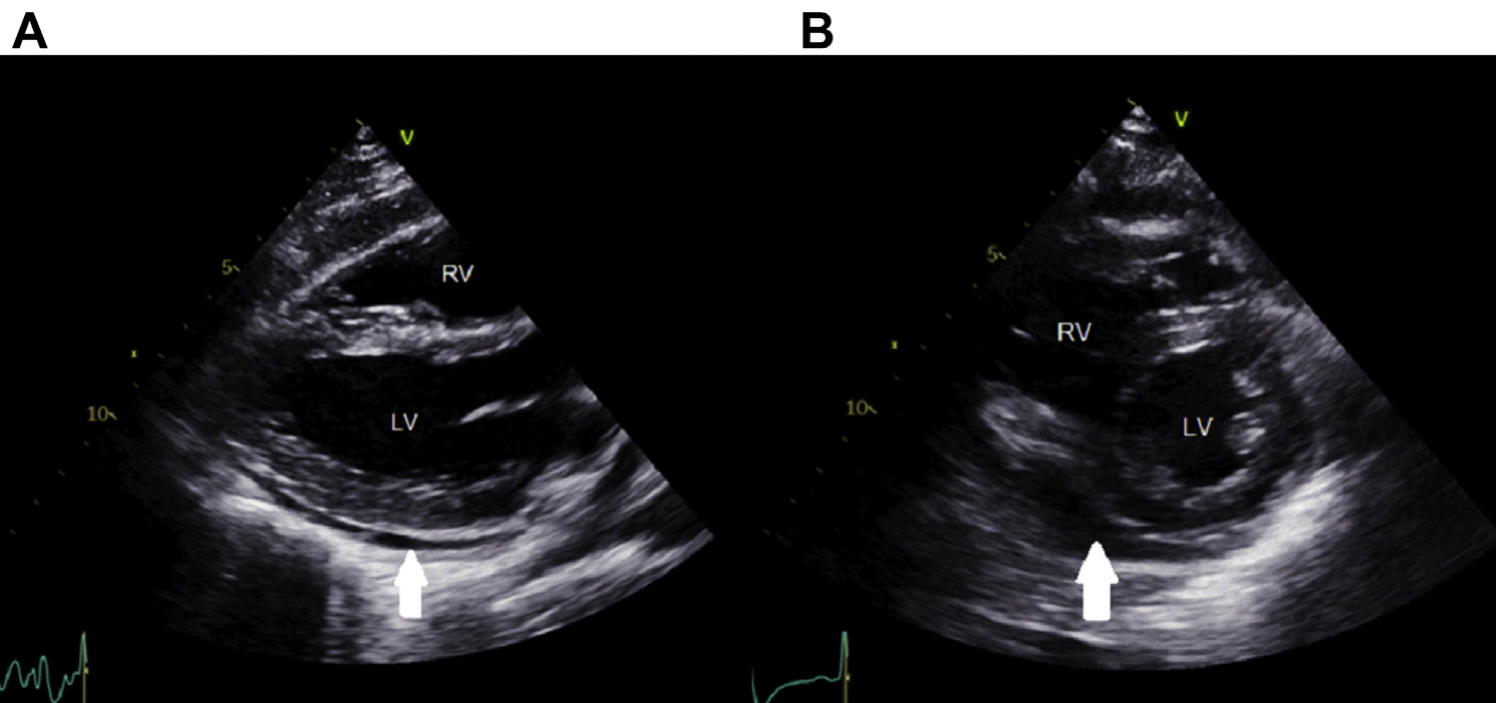
Spontaneous Improvement on Preprocedural Echocardiography **(A)** Parasternal long-axis and **(B)** parasternal short-axis views repeated 45 minutes after the previous echocardiogram. Spontaneous and significant improvement in the size of the pericardial effusion was noted **(arrows).** Abbreviations as in Figure 1.

Given his clinical deterioration, a transthoracic echocardiogram was repeated to rule out cardiac tamponade, and it showed a moderate to large pericardial effusion, clearly larger within 24 hours (Figures 3A to 3C, Videos 2A, 2B, and 2C). The inferior vena cava (IVC) was plethoric and noncollapsing. No cardiac chamber collapse was noted. Given the decreased BP, tachycardia, and rapid progression in size of the effusion, urgent pericardiocentesis was requested. However, preprocedural echocardiographic images taken approximately 45 minutes after the aforementioned study revealed only a small pericardial effusion (Figures 4A and 4B, Videos 3A and 3B). There was concomitant improvement in BP and resolution of the tachycardia. The evanescent nature of the effusion was not readily explainable.

The patient was admitted to the cardiovascular intensive care unit. Ibuprofen was replaced by prednisone 50 mg daily because an acute kidney injury developed. After 9 days of treatment with prednisone, his symptoms persisted, but there was a reduction in the inflammatory markers and leukocyte count, with a C-reactive protein (CRP) level of 17.8 mg/L, down from a CRP level of 186.9 mg/L on presentation.

**Figure 5:**
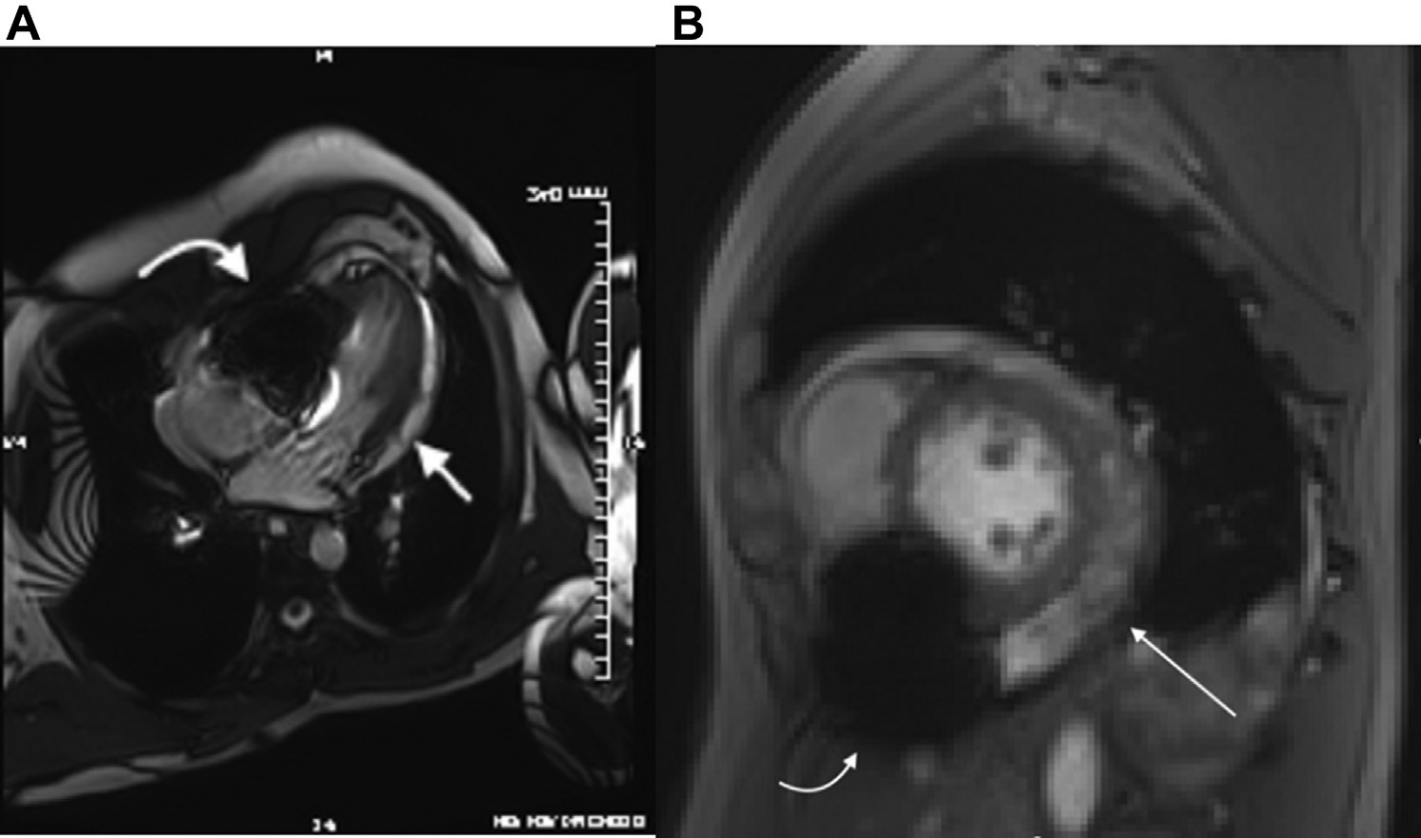
Cardiac Magnetic Resonance Steady-state free precession bright blood cardiac magnetic resonance images in **(A)** 4-chamber and **(B)** short-axis planes demonstrating the pericardial effusion **(straight arrow)** and a large area of susceptibility artifact with signal loss and distortion at the bottom of the right ventricle **(curved arrow).**

**Figure 6:**
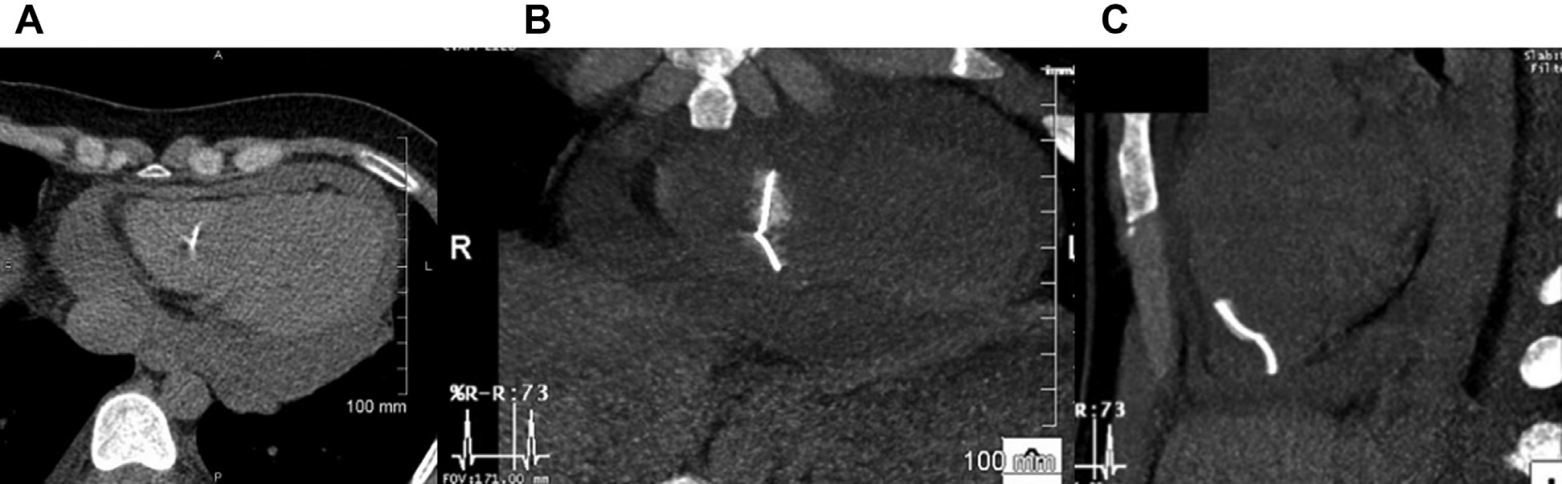
Cardiac Computed Tomography Noncontrast computed tomography (CT) images demonstrating **(A)** circumferential pericardial effusion and a linear metal density in the right ventricle. **(B and C)** Curvilinear reformatted images depicting the arrangement of 2 adjacent needles or 1 fractured needle with 1 end anchored to the right ventricular wall. R = right.

To characterize the pericardium more clearly, a cardiac magnetic resonance (CMR) scan was ordered. It demonstrated a moderate, circumferential pericardial effusion with associated late gadolinium enhancement and diffuse pericardial thickening. A large area of unexplained susceptibility blooming artifact obscuring the right ventricle motivated additional investigation (Figures 5A and 5B, Videos 4A and 4B). Computed tomography revealed the presence of 2 linear metallic fragments originating within the right ventricular myocardium and protruding into the right ventricular cavity (Figures 6A to 6C).

On repeated questioning, the patient admitted to undergoing acupuncture for chronic indigestion 3 months earlier in South Korea. At that time, acupuncture needles were inserted into the skin of the anterior chest and upper abdomen but were presumed to be removed at the end of the session. Given the imaging and clinical constellation, a diagnosis of retained acupuncture needles piercing the inferior aspect of the right ventricle, by direct migration, and consequent hemopericardium was entertained.

## Management

**Figure 7:**
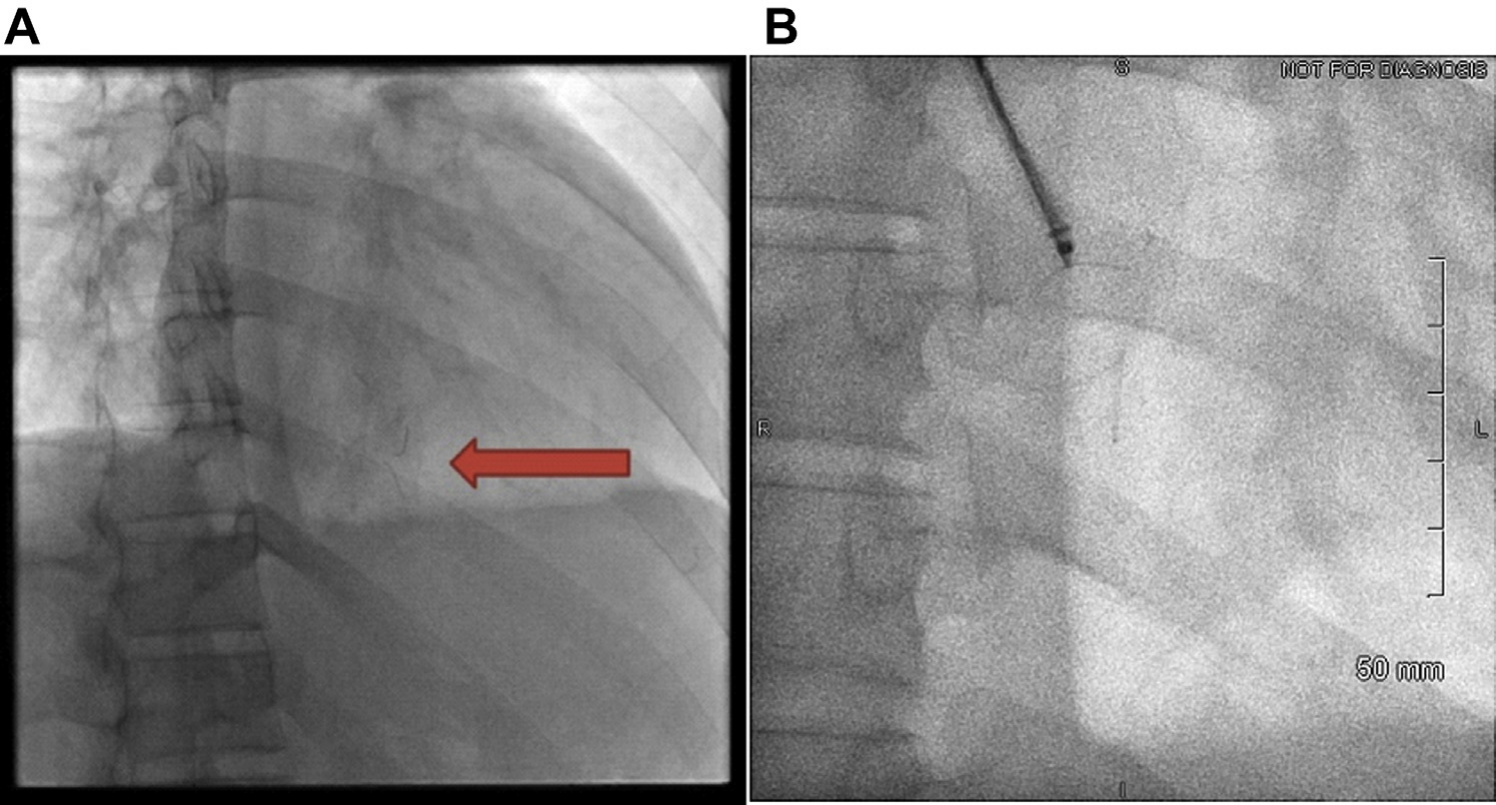
Fluoroscopic Confirmation of Acupuncture Needle and Removal Fluoroscopic images demonstrating **(A)** 2 hair-thin linear objects projecting to the heart **(arrow)** and **(B)** an endovascular snare capturing 1 of the objects, before collapsing it into the vascular sheath.

**Figure 8:**
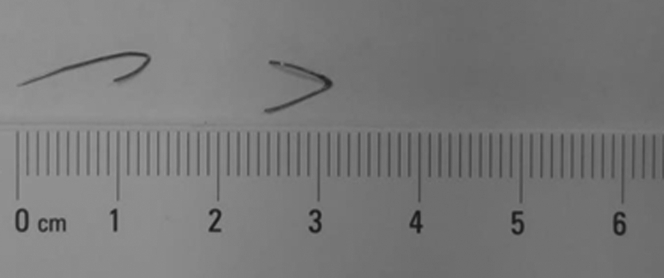
Retrieved Metallic Fragments Metallic fragments retrieved endovascularly from the right ventricle.

After discussion with cardiac surgery and interventional radiology, it was decided to attempt percutaneous retrieval by interventional radiology. Chest fluoroscopy confirmed the presence of 2 metallic fragments in the inferior region of the heart (Figures 7A and 7B, Video 5). Right jugular venous access was obtained with insertion of a 7-F Flexor Ansel angled vascular sheath (Cook Medical). The 12- to 20-mm EN-Snare endovascular system (Merit Medical) was used to capture each metallic fragment individually under fluoroscopic guidance. The fragments were then pulled into the sheath, thereby bending their shape into a “U.” Needle fragments, measuring 9 and 7 mm in length, were successfully extracted (Figure 8).

## Discussion

Acupuncture is a popular alternative therapy commonly used for musculoskeletal symptoms. Although generally considered safe, insertion of acupuncture needles into the chest is associated with adverse outcomes such as pneumothorax (1). A recent systematic review identified 30 cases of adverse cardiovascular events resulting from acupuncture (2). In this group, 8 patients had infectious complications (mostly infective endocarditis), and 22 patients had cardiac tamponade. Onset of symptoms can occur up to a decade after acupuncture (3). In this case, the delay to symptom onset was approximately 3 months.

With respect to the resolving pericardial effusion, we postulated that the needle may have shifted over time, thus creating an iatrogenic fistula between the pericardial cavity and the left pleural space, into which the effusion likely drained.

We opted to try a less invasive approach in a young, healthy adult without comorbidities, who was clinically stable, but symptomatic, to avoid the sequelae and potential complications of cardiac surgery. The decision was anchored by a case report detailing success with a percutaneous technique, as well as the radiologist’s previous experience retrieving embolized IVC filter fragments by a transjugular approach (4).

This case highlights the importance of searching for nonidiopathic causes when the condition of a patient with pericarditis deteriorates rapidly after initiation of usual therapy. It also emphasizes the complementary role of multimodality imaging in evaluating such cases.

## Follow-up

The patient’s symptoms persisted despite removal of the needle fragments and a course of prednisone for 4 weeks. Consequently, anakinra was initiated, and dosing frequency was tapered over the course of 10 months until complete symptom resolution. A repeat CMR scan after 6 months demonstrated interval resolution of the pericardial effusion, with no residual abnormalities.

## Conclusions

Pericarditis that worsens rapidly with initial therapy should trigger investigation for a nonidiopathic cause. Although rare, cardiac complications of acupuncture, including pericardial effusions and cardiac perforation, have been reported. In the right setting, transvenous retrieval of intracavitary foreign bodies, such as needles, appears to be feasible and safe.

## Funding Support and Author Disclosures

The authors have reported that they have no relationships relevant to the contents of this paper to disclose.

## Appendix

Supplemental Video 1A Initial Echocardiography. **(A)** Parasternal long-axis, **(B)** parasternal short-axis, and **(C)** off-axis apical views on transthoracic echocardiography showing a small pericardial effusion.

Supplemental Video 1B Initial Echocardiography. **(A)** Parasternal long-axis, **(B)** parasternal short-axis, and **(C)** off-axis apical views on transthoracic echocardiography showing a small pericardial effusion.

Supplemental Video 1C Initial Echocardiography. **(A)** Parasternal long-axis, **(B)** parasternal short-axis, and **(C)** off-axis apical views on transthoracic echocardiography showing a small pericardial effusion.

Supplemental Video 2A Repeated Echocardiography. Repeat **(A)** parasternal long-axis, **(B)** parasternal short-axis, and **(C)** off-axis apical views. The pericardial effusion has increased in size.

Supplemental Video 2B Repeated Echocardiography. Repeat **(A)** parasternal long-axis, **(B)** parasternal short-axis, and **(C)** off-axis apical views. The pericardial effusion has increased in size.

Supplemental Video 2C Repeated Echocardiography. Repeat **(A)** parasternal long-axis, **(B)** parasternal short-axis, and **(C)** off-axis apical views. The pericardial effusion has increased in size.

Supplemental Video 3A Spontaneous Improvement on Preprocedural Echocardiography. **(A)** Parasternal long-axis and **(B)** parasternal short-axis views repeated 45 minutes after the previous echocardiogram. Spontaneous and significant improvement in the size of the pericardial effusion was noted.

Supplemental Video 3B Spontaneous Improvement on Preprocedural Echocardiography. **(A)** Parasternal long-axis and **(B)** parasternal short-axis views repeated 45 minutes after the previous echocardiogram. Spontaneous and significant improvement in the size of the pericardial effusion was noted.

Supplemental Video 4A 4-Chamber Plane on CMR. Steady-state free precession bright blood cardiac magnetic resonance images in the **(A)** 4-chamber plane and **(B)** short-axis plane demonstrating a large area of susceptibility artifact with signal loss and distortion at the bottom of the right ventricle.

Supplemental Video 4B 4-Chamber Plane on CMR. Steady-state free precession bright blood cardiac magnetic resonance images in the **(A)** 4-chamber plane and **(B)** short-axis plane demonstrating a large area of susceptibility artifact with signal loss and distortion at the bottom of the right ventricle.

Supplemental Video 5 Fluoroscopic Confirmation of Acupuncture Needle and Removal. Fluoroscopic images demonstrating endovascular snare capturing 1 of the needle fragments.
